# Real-world prospective analysis of treatment patterns in durvalumab maintenance after chemoradiotherapy in unresectable, locally advanced NSCLC patients

**DOI:** 10.1007/s10637-021-01091-9

**Published:** 2021-03-11

**Authors:** Julian Taugner, Lukas Käsmann, Chukwuka Eze, Alexander Rühle, Amanda Tufman, Niels Reinmuth, Thomas Duell, Claus Belka, Farkhad Manapov

**Affiliations:** 1 Department of Radiation Oncology, University Hospital, LMU Munich, Marchioninistrasse 15, 81377 Munich, Germany; 2 Comprehensive Pneumology Center Munich (CPC-M), Member of the German Center for Lung Research (DZL), Munich, Germany; 3 German Cancer Consortium (DKTK), Munich, Germany; 4 Department of Radiation Oncology, Freiburg University Medical Center, Freiburg, Germany; 5 German Cancer Consortium (DKTK) Partner Site Freiburg, German Cancer Research Center (DKFZ), Heidelberg, Germany; 6 Division of Respiratory Medicine and Thoracic Oncology, Department of Internal Medicine V, Thoracic Oncology Centre Munich, LMU Munich, Munich, Germany; 7 Asklepios Kliniken GmbH, Asklepios Fachkliniken Muenchen, Gauting, Germany

**Keywords:** Treatment pattern, PD-L1 inhibitor, Chemoradiotherapy, Checkpoint inhibition, Non-small cell lung cancer

## Abstract

The aim of this prospective study is to evaluate the clinical use and real-world efficacy of durvalumab maintenance treatment after chemoradiotherapy (CRT) in unresectable stage, locally advanced non-small cell lung cancer (NSCLC). All consecutive patients with unresectable, locally advanced NSCLC and PD-L1 expression (≥1%) treated after October 2018 were included. Regular follow up, including physical examination, PET/CT and/or contrast-enhanced CT-Thorax/Abdomen were performed every three months after CRT. Descriptive treatment pattern analyses, including reasons of discontinuation and salvage treatment, were undertaken. Statistics were calculated from the last day of thoracic irradiation (TRT). Twenty-six patients were included. Median follow up achieved 20.6 months (range: 1.9–30.6). Durvalumab was initiated after a median of 25 (range: 13–103) days after completion of CRT. In median 14 (range: 2–24) cycles of durvalumab were applied within 6.4 (range 1–12.7) months. Six patients (23%) are still in treatment and seven (27%) have completed treatment with 24 cycles. Maintenance treatment was discontinued in 13 (50%) patients: 4 (15%) patients developed grade 3 pneumonitis according to CTCAE v5 after a median of 3.9 (range: 0.5–11.6) months and 7 (range: 2–17) cycles of durvalumab. Four (15%) patients developed grade 2 skin toxicity. One (4%) patient has discontinued treatment due to incompliance. Six and 12- month progression-free survival (PFS) rates were 82% and 62%, median PFS was not reached. No case of hyperprogression was documented. Eight (31%) patients have relapsed during maintenance treatment after a median of 4.8 (range: 2.2–11.3) months and 11 (range: 6–17) durvalumab cycles. Two patients (9%) developed a local-regional recurrence after 14 and 17 cycles of durvalumab. Extracranial distant metastases and brain metastases as first site of failure were detected in 4 (15%) and 2 (8%) patients, respectively. Three (13%) patients presented with symptomatic relapse. Our prospective study confirmed a favourable safety profile of durvalumab maintenance treatment after completion of CRT in unresectable stage, locally advanced NSCLC in a real-world setting. In a median follow-up time of 20.6 months, durvalumab was discontinued in 27% of all patients due to progressive disease. All patients with progressive disease were eligible for second-line treatment.

## Introduction

Lung cancer is the leading cause of malignancy-related mortality [1]. Unresectable and locally advanced non-small cell lung cancer (NSCLC) is associated with a poor local and distant control resulting in a unfavorable survival [2–6]. In the last two years, durvalumab maintenance treatment after definitive chemoradiotherapy (CRT) represents the new multi-modal standard approach for inoperable stage III non-small cell lung cancer (NSCLC). The pivotal approval trial (PACIFIC) demonstrated an unprecedented improvement of progression-free (PFS) and overall survival (OS) in the durvalumab compared to the placebo arm [7–9]. The study also demonstrated a low toxicity profile of durvalumab. Severe (grade ≥ 3) adverse events were not significantly increased in the experimental arm (34.9%) compared to the control arm (31.1%) and low rates of severe pneumonitis (grade ≥ 3) were reported.

Unfortunately, comprehensive radiation treatment planning data including irradiated tumor volumes and involved lymph-node stations are not yet available. However, patients who did not meet planning criteria (MLD < 20Gy, total lung volume receiving 20Gy < 35% and total heart volume receiving 50Gy < 25%) and patients with grade ≥ 2 pneumonitis after completion of TRT were excluded from the study.Generally, chemoimmunotherapy as well as durvalumab maintenance treatment after CRT has been rapidly implemented in clinical routine [3, 4]. Our survey investigating the implementation of durvalumab maintenance treatment in the German Society of Radiation Oncology (DEGRO e.V.) found that durvalumab maintenance is already administered by the majority of respondents [10].

To date, there is little data regarding treatment pattern and efficacy of durvalumab maintenance treatment after CRT in the real-world setting. Here we present data on maintenance durvalumab including pattern of failure and treatment interruption.

## Patients and method

This prospective study included twenty-six consecutive patients who received concurrent or sequential conventionally fractionated CRT with consolidation durvalumab for unresectable and locally advanced NSCLC between 2018 and 2020. The local ethics committee granted approval to conduct this study (17–230) and all patients gave their informed consent for participation.

All patients were treated at a single tertiary cancer center and enrolled if eligible for platinum-based chemoradiotherapy followed by durvalumab maintenance treatment. PD-L1 negative patients or patients with autoimmune disorders were excluded. As part of the pre-treatment work-up, radiographic imaging was performed using positron emission tomography (PET)-CT in 25 (96%) patients and computed tomography (CT) for 1 (4%) patient. Cranial contrast-enhanced magnet resonance imaging (MRI) was performed in 23 (89%) patients, while the others received a contrast-enhanced head CT. PD-L1 expression was assessed per VENTANA PD-L1 (SP263) Assay (Roche Diagnostics, F. Hoffmann-La Roche Ltd., Basel, Switzerland). All patients were discussed prior to treatment at the multidisciplinary tumor board and all patients were deemed unresectable by an experienced group of thoracic surgeons, pulmonologists and radiation oncologists. During the course of treatment and prior to application of durvalumab, complete blood work was performed. In addition, pulmonary function tests were performed routinely every 3 months.

### Chemoradiotherapy

Thoracic radiotherapy (TRT) was planned and delivered with arms positioned overhead in a WingSTEP™ (Innovative Technologie Völp, Innsbruck, Austria) in supine position. If patients received induction chemotherapy, only the residual primary tumor volume was contoured but all initially involved lymph-node stations were included in the planning target volume (PTV). The target volumes were defined according to the Advisory Committee in Radiation Oncology Practice (ACROP) of the European Society for Radiotherapy and Oncology (ESTRO) guidelines [11]. To generate the PTV, a margin of 6 mm (axial) and 9 mm (cranio-caudal) was added to the clinical target volume (CTV). Radiation was delivered on a Linear Accelerator (LINAC) with megavoltage capability (6–15 MV) using Volumetric Modulated Arc Therapy (VMAT) in all patients. Inter-fraction motion was routinely assessed on cone-beam CT.

After CRT and a follow-up CT scan, durvalumab was administered intravenously at a dose of 10 mg/kg every two weeks for up to 24 cycles until progression or unacceptable toxicity according to the Common Terminology Criteria of Adverse Events Version 5 (CTCAE v5).

### Patient follow-up

CT/PET-CT scans, routine blood work, pulmonary function testing and clinical examinations were performed every 3 months during durvalumab treatment; contrast-enhanced brain MRI, bone-scintigraphy and bronchoscopy were only performed if clinically indicated.

Local-regional recurrence (LRR) along with new distant metastases (DM) and brain metastasis (BM) were documented with CT, PET-CT and MRI scans. Histological or cytological confirmation of progressive disease was not obligatory. Median follow-up was calculated as the time from the last day of TRT to last follow-up or loss of follow-up. Progression-free survival (PFS) was defined as the time from end of TRT until disease progression or death. Similarly, overall survival (OS) was assessed from the end of TRT. All statistics were performed using IBM SPSS version 26 (IBM, Armonk, New York, USA).

## Results

### Cohort characteristics

Patients characteristics can be found in Table 1. Median follow up after CRT was 20.6 months (range: 1.9–30.6). Median age at diagnosis was 67.6 (range: 43.8–78.4) years, 16 (62%) patients were older than 65 years and 9 (35%) patients were female. Ten (39%) patients were diagnosed with squamous cell carcinomas and 12 (46%) with adenocarcinomas, 4 (15.4%) were classified as Non-otherwise specified (NOS). Twenty-four (92%) were irradiated to a total dose of at least 60Gy in equivalent dose in 2Gy fractions (EQD2). Thirteen (50%) patients were diagnosed with UICC stage IIIB and 3 (12%) with UICC IIIC disease. One patient had initial UICC stage IV with M1b disease (cervical lymph node metastases) treated in curative intention. Overexpression of PD-L1 (≥ 50%) on tumor cells was detected in 12 (46%) patients. Fourteen (54%) patients were treated with induction chemotherapy. All but one patient (96%) received two cycles of concomitant platinum-based doublet-chemotherapy. Regarding TRT, median PTV amounted to 680.3 cc (range: 204.5–1234.5 cc). The corresponding planning criteria (mean lung dose <20Gy and total lung volume irradiated with 20Gy < 35%) were fulfilled in all cases [Table 1].

**Table 1:** 55 characteristics

	**N (%)**
**Total**	26 (100)
**Age**	
median years	67.6
> 65 years	16 (61.5)
**Gender**	
Male	17 (65.4)
Female	9 (34.6)
**T-stage**	
1	1 (3.8)
2	5 (19.2)
3	8 (30.8)
4	12 (46.2)
**N-stage**	
0	5 (19.2)
1	1 (3.8)
2	14 (53.8)
3	6 (23.1)
**M 1**	1 (3.8)
**UICC 8th edition**	
IIIA	9 (34.6)
IIIB	13 (50.0)
IIIC	3 (11.5)
IVA	1 (3.8)
**PTV-size**	
median cc	680.3
≥ 700 cc	12 (46.2)
**Histology**	
Squamous cell carcinoma (SCC)	10 (38.5)
Adenocarcinoma (AC)	12 (46.2)
Not otherwise specified (NOS)	4 (15.4)
**Radiographic imaging**	
PET-CT	25 (96.2)
cMRI	23 (88.5)
**Treatment**	
Concurrent chemoradiation (CRT)	25 (96.2)
Induction chemotherapy	14 (53.8)
**Median-FU** months after CRT	20.6
**OS**	
6-months	100%
12-months	100%
**PFS**	
6-months	82%
12-months	62%

### Durvalumab treatment and reasons for treatment interruption

All patients received a contrast-enhanced CT thorax/abdomen after a median of 10 days after completion of CRT, in which none had shown progression or features suggestive of severe pneumonitis. Durvalumab was initiated at a median of 25 (range: 13–103) days after the end of CRT. A median of 14 (range: 2–24) cycles of durvalumab were applied within 6.4 (range 1–12.7) months. Seven (27%) patients have completed treatment with 24 cycles and six patients (23%) are still on treatment.

**Fig. 1:**
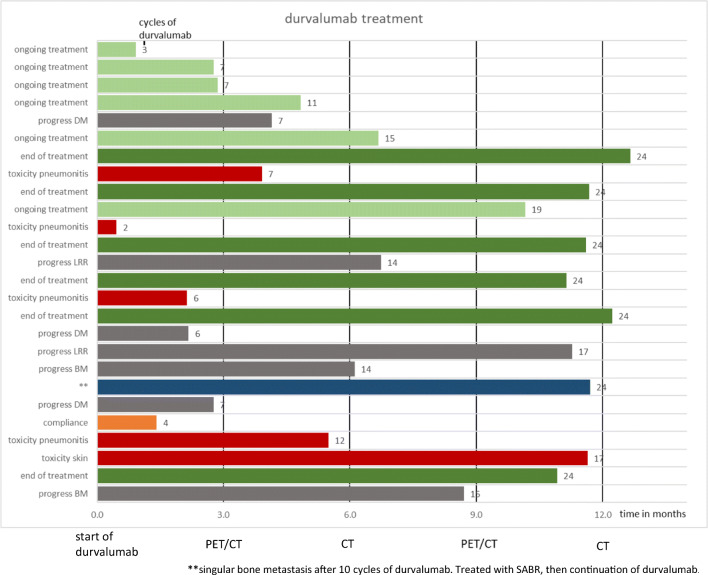
Overview about durvalumab treatment

Maintenance treatment was discontinued in 13 (50%) patients: 4 (15%) patients developed grade 3 toxicity according to CTCAE v5 after a median of 3.9 (range: 0.5–11.6) months and 7 (range: 2–17) cycles of durvalumab. All these patients presented with CTC grade 3 pneumonitis and no toxicity ≥ grade 3 of another origin was documented. Three patients with grade 3 pneumonitis recovered after interruption of treatment and high dose corticosteroid therapy. One patient started with long-term oxygen therapy. Four (15%) patients developed skin toxicity grade 2 with itchy, red, swollen, and cracked skin similar to atopic dermatitis. In one of these patients (4%), treatment was interrupted because of skin superinfection with Methicillin-resistant *Staphylococcus aureus*. No patients suffered from gastrointestinal toxicity in the present cohort. Eight (35%) patients relapsed during maintenance treatment after a median of 4.8 (range: 2.2–11.3) months and 11 (range: 6–17) durvalumab cycles. One (4%) patient has discontinued treatment due to incompliance after 1.4 months and 4 cycles of durvalumab [Fig. 1].

### Pattern of failure during durvalumab maintenance

**Fig. 2:**
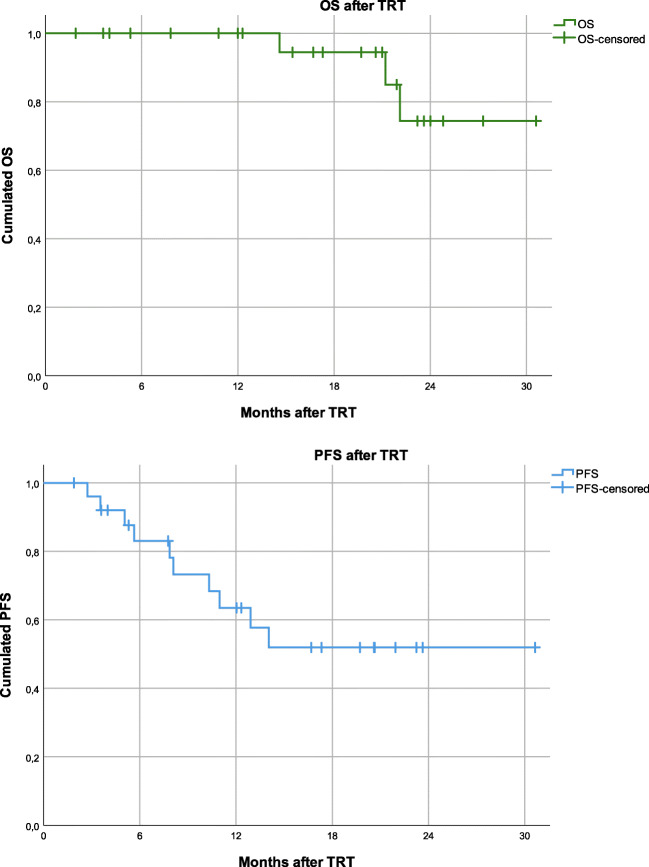
OS and after the last day of TRT

Three patients (11.5%) have died after a median of 21.2 months after TRT. Median PFS was not reached. Six and 12- month PFS rates were 82% and 62% [Fig. 2]. No case of hyperprogression was documented. Two patients (8%) developed LRR 8 / 13 months after CRT and 14 / 17 cycles of durvalumab. Extracranial DMs and BMs as a first site of failure were detected in 4 (15%) and 2 (8%) patients after a median of 7 and 14 cycles of durvalumab and a median time of 4 and 9 months after CRT, respectively. Three (12%) patients presented with symptomatic relapse (two of these patients had BM first), whereas asymptomatic progression was detected via after-care imaging in 7 (27%) patients.

All patients with progressive disease after durvalumab were eligible for second-line therapy: One patient (50%) with local-regional recurrence was treated with re-irradiation and the other patient (50%) received the tyrosine kinase inhibitor LOXO-292 targeting the RET proto-oncogene. Surgical resection of symptomatic brain metastasis was performed in one patient (50%), followed by stereotactic fractionated radiation to the resection cavity. The other patient with BM first received stereotactic radiosurgery for two brain metastases. One patient (25%) with DM-first received stereotactic ablative body radiotherapy (SABR) to a singular bone-lesion discovered after 10 cycles of durvalumab and continued durvalumab without any further relapse completing 24 cycles. One patient (25%) with early onset of multiple bone and lymph node metastasis after 6 cycles of durvalumab (2.7 months after TRT) received second line chemotherapy with docetaxel and nintedanib. One patient (25%) with multiple metastasis after 7 cycles of durvalumab (3.6 months after TRT) was treated with carboplatin and pemetrexed. One patient (25%) with bone metastasis (7 cycles of durvalumab, 5.7 months after TRT) received palliative radiotherapy [Table 2].

**Table 2:** Progression during durvalumab maintenance and its treatment

Stage	T-Stage	N-Stage	Histology	PD-L1%	Site of Progression	Progression months after TRT	Cylcles of durva-lumab applied	Months of durvalumab treatment	Oligo-progression	Therapy after progression
IIIC	3	3	AC	1	DM multi	2.7	6	2.2	no	Docetaxel + Nintedanib
IIIC	4	3	AC	90	DM bone	3.6	7	2.8	no	Carboplatin + Pemetrexed
IIIB	4	2	NOS	30	DM bone	5.7	7	4.1	yes	palliative radiotherapy (10x 3Gy)
IIIB	2	3	SCC	80	DM sing. Bone	5.1	10	4.1	yes	SABR (10 x 4Gy) + continuation of durvalumab treatment
IIIA	2	2	AC	30	BM	7.9	14	6.1	yes	stereotactic radiosurgery (SRS, 20Gy Iso 80%)
IIIA	1	2	AC	15	LRR	8.1	14	6.7	yes	RET-TKI (LOXO-292)
IIIB	3	2	AC	40	BM	11.0	15	8.7	yes	surgery + radiotherapy (SRS, 5x 5Gy Iso 80%)
IIIB	4	2	SCC	90	LRR	14.0	17	11.3	yes	re-radiotherapy with SABR (10x 4Gy)

Progression occurred in two patients which interrupted durvalumab due to toxicity, no case of progression was observed in patients completing durvalumab maintenance. Median survival of patients with progressive disease (*n* = 10) after onset of progression was 18.6 (95%CI: 10.9–26.2) months.

Out of four patients with treatment interruption due to CTCAE °III pneumonitis, two patients (50%) had LRR 3.9 and 11.9 months after the last cycle of durvalumab (10.3 and 12.3 months after TRT) and two patients are alive and without relapse 17.3 and 30.6 months after TRT.

## Discussion

This prospective report provides an overview of treatment patterns and utility of durvalumab maintenance treatment after completion of CRT in a high-volume tertiary cancer center. Since approval of durvalumab from the European Medicines Agency (EMA) in September 2018, twenty-six consecutive patients with PD-L1 expressing unresectable stage III – IVA NSCLC were treated and included in the analysis. Based on tumor characteristics including the UICC stage and high PTV volumes, our patient cohort is at high risk of treatment-related toxicity [12, 13]. Nevertheless, after objective evaluation of response with contrast-enhanced CT thorax/abdomen after completion of CRT, all patients started durvalumab maintenance therapy.

Preliminary results confirm a high efficacy of durvalumab in this real-life patient cohort with median PFS not reached, 12-month PFS rate of 62% and 12-month OS rate of 100% after a median follow-up time of 20.6 months after the end of TRT. Our results also confirm a favourable safety profile of this maintenance therapy without any grade 4/5 toxicity and 15% grade 3 adverse events (4/26 patients) per CTCAE v5. Pneumonitis (15%, 4 patients) was the predominant toxicity resulting in treatment interruption. All these findings are in close accordance with recently published data [14–24].

Patient compliance was very high (see Fig. 1) and patients were closely monitored. Compared with historical studies of chemotherapy or targeted consolidation therapy, these results seem advantageous. For example, the Hoosier Oncology Group and U.S. Oncology phase III trial on docetaxel consolidation after CRT with cisplatin and etoposide reported 10.9% febrile neutropenia (grade ≥ 3), grade 3 to 5 pneumonitis in 9.6% and 5.5% fatal toxicity [25]. In the PROCLAIM trial (Pemetrexed-Cisplatin or Etoposide-Cisplatin Plus Thoracic Radiation Therapy Followed by Consolidation Chemotherapy), high rates of grade 3–4 neutropenia and other grade 3–4 toxicity were observed especially in the standard therapy arm with concomitant cisplatin and etoposide followed by two cycles of consolidation platinum-based doublet chemotherapy [26]. A German phase III study (GILT) on consolidation with two cycles of cisplatin and oral vinorelbine after CRT revealed no fatal toxicity but a higher incidence of haematological toxicity: 26.7% and 22.1% had grade 3 or 4 leukopenia and neutropenia during consolidation treatment and one patient developed febrile neutropenia [27].

Another phase III trial of maintenance gefitinib after docetaxel consolidation and CRT (SWOG S0023) reported a mortality rate of 2% during CRT, 4% during docetaxel consolidation and 2% for gefitinib maintenance vs 0% for placebo [28]. Grade 4 neutropenia was observed in 33% of patients during docetaxel consolidation. Pneumonitis (≥ grade 3) after CRT during docetaxel consolidation was observed in 7% of patients (1% grade 5) and during gefitinib maintenance in 3% of patients [28].

Regarding patterns of failure, we observed a similar rate of LRR and distant relapses (Table 1). Altogether, durvalumab was interrupted in 7/26 (27%) patients due to disease progression. Importantly, no case of hyperprogression was observed and no recurrences were detected in the first three months of maintenance therapy. Three patients were diagnosed with asymptomatic DM on the first restaging PET/CT during durvalumab. Generally, distant relapse appear to occur earlier (median 6 months) than local-regional recurrence (7 and 13 months) after CRT. Interestingly asymptomatic relapse discovered during routine PET/CT or CT-scans was more frequently diagnosed than symptomatic relapse (5 and 3 cases, respectively).

Of importance is the fact that all relapsed patients were started on second-line therapy or continued with durvalumab after SABR. Overall, there was no observable deterioration of performance status (PS), all but one patient with grade 3 pneumonitis recovered completely after high dose corticosteroid therapy. This can influence the efficacy and duration of salvage treatment as good and stable PS was previously shown to be an important prognostic factor [29, 30].

Given the limitations of this small, prospective study and the relatively short median follow-up period, durvalumab maintenance treatment appears to be safe and effective in patients with PD-L1 expressing locally advanced NSCLC after CRT. The observed advantageous toxicity profile is in close accordance with previous results of the PACIFIC study. Neither hyperprogression nor early deaths during treatment were seen, and the low number of symptomatic relapses as well as high treatment compliance confirm the suitability of this treatment for real-world clinical use.

## Conclusion

Our prospective study confirmed a favourable safety profile of durvalumab maintenance treatment after completion of CRT in locally advanced and PD-L1 expressing NSCLC patients in a real-world setting. In a median follow-up time of 20.6 months, durvalumab was discontinued in 27% of all patients due to progressive disease. All patients with progressive disease were eligible for second-line treatment.

## Data Availability

The data that support the findings of this study are available from the corresponding author, upon reasonable request.

## References

[1] Siegel RL, Miller KD, Jemal A (2019). Cancer statistics, 2019. CA A Cancer J Clin.

[2] Taugner J, Eze C, Käsmann L, Roengvoraphoj O, Gennen K, Karin M, Petrukhnov O, Tufman A, Belka C, Manapov F (2020). Pattern-of-failure and salvage treatment analysis after chemoradiotherapy for inoperable stage III non-small cell lung cancer. Radiat Oncol.

[3] Alexander M, Wolfe R, Ball D, Conron M, Stirling RG, Solomon B, MacManus M, Officer A, Karnam S, Burbury K, Evans SM (2017). Lung cancer prognostic index: a risk score to predict overall survival after the diagnosis of non-small-cell lung cancer. Br J Cancer.

[4] Yoon SM, Shaikh T, Hallman M (2017). Therapeutic management options for stage III non-small cell lung cancer. World J Clin Oncol.

[5] Dillman RO, Herndon J, Seagren SL, Eaton WL, Green MR (1996). Improved survival in stage III non-small-cell lung cancer: seven-year follow-up of cancer and leukemia group B (CALGB) 8433 trial. J Natl Cancer Inst.

[6] Machtay M, Paulus R, Moughan J, Komaki R, Bradley JE, Choy H, Albain K, Movsas B, Sause WT, Curran WJ (2012). Defining local-regional control and its importance in locally advanced non-small cell lung carcinoma. J Thorac Oncol.

[7] Antonia SJ, Villegas A, Daniel D, Vicente D, Murakami S, Hui R, Yokoi T, Chiappori A, Lee KH, de Wit M, Cho BC, Bourhaba M, Quantin X, Tokito T, Mekhail T, Planchard D, Kim YC, Karapetis CS, Hiret S, Ostoros G, Kubota K, Gray JE, Paz-Ares L, de Castro Carpeño J, Wadsworth C, Melillo G, Jiang H, Huang Y, Dennis PA, Özgüroğlu M (2017). Durvalumab after Chemoradiotherapy in stage III non-small-cell lung Cancer. N Engl J Med.

[8] Antonia SJ, Villegas A, Daniel D, Vicente D, Murakami S, Hui R, Kurata T, Chiappori A, Lee KH, de Wit M, Cho BC, Bourhaba M, Quantin X, Tokito T, Mekhail T, Planchard D, Kim YC, Karapetis CS, Hiret S, Ostoros G, Kubota K, Gray JE, Paz-Ares L, de Castro Carpeño J, Faivre-Finn C, Reck M, Vansteenkiste J, Spigel DR, Wadsworth C, Melillo G, Taboada M, Dennis PA, Özgüroğlu M (2018). Overall survival with Durvalumab after Chemoradiotherapy in stage III NSCLC. N Engl J Med.

[9] Gray JE, Villegas A, Daniel D, Vicente D, Murakami S, Hui R, Kurata T, Chiappori A, Lee KH, Cho BC, Planchard D, Paz-Ares L, Faivre-Finn C, Vansteenkiste JF, Spigel DR, Wadsworth C, Taboada M, Dennis PA, Özgüroğlu M, Antonia SJ (2020). Three-year overall survival with Durvalumab after Chemoradiotherapy in stage III NSCLC-update from PACIFIC. J Thorac Oncol.

[10] Käsmann L, Eze C, Taugner J, Roengvoraphoj O, Belka C, Manapov F (2020). Implementation of durvalumab maintenance treatment after concurrent chemoradiotherapy in inoperable stage III non-small cell lung cancer (NSCLC)-a German radiation oncology survey. Transl. Lung Cancer Res..

[11] Nestle U, de Ruysscher D, Ricardi U, Geets X, Belderbos J, Pöttgen C, Dziadiuszko R, Peeters S, Lievens Y, Hurkmans C, Slotman B, Ramella S, Faivre-Finn C, McDonald F, Manapov F, Putora PM, LePéchoux C, van Houtte P (2018). ESTRO ACROP guidelines for target volume definition in the treatment of locally advanced non-small cell lung cancer. Radiother Oncol.

[12] Käsmann L, Dietrich A, Staab-Weijnitz CA, Manapov F, Behr J, Rimner A, Jeremic B, Senan S, de Ruysscher D, Lauber K, Belka C (2020). Radiation-induced lung toxicity - cellular and molecular mechanisms of pathogenesis, management, and literature review. Radiat Oncol.

[13] Hosoya K, Fujimoto D, Kawachi H, Sato Y, Kogo M, Nagata K, Nakagawa A, Tachikawa R, Hiraoka S, Kokubo M, Tomii K (2019). Ineligibility for the PACIFIC trial in unresectable stage III non-small cell lung cancer patients. Cancer Chemother Pharmacol.

[14] Shaverdian N, Thor M, Shepherd AF, Offin MD, Jackson A, Wu AJ, Gelblum DY, Yorke ED, Simone CB, Chaft JE (2020). Radiation pneumonitis in lung cancer patients treated with chemoradiation plus durvalumab. Cancer Med.

[15] Vansteenkiste J, Naidoo J, Faivre-Finn C, Özgüroğlu M, Villegas A, Daniel D, Murakami S, Hui R, Lee K, Cho BC, Kubota K, Poole L, Wadsworth C, Dennis P, Antonia S (2018). MA05.02 PACIFIC Subgroup Analysis: Pneumonitis in Stage III, Unresectable NSCLC Patients Treated with Durvalumab vs. Placebo After CRT. J Thorac Oncol.

[16] Vansteenkiste JF, Naidoo J, Faivre-Finn C, Özgüroğlu M, Villegas A, Daniel D, Murakami S, Hui R, Lee KH, Cho BC, Kubota K, Taboada M, Wadsworth C, Dennis PA, Antonia SJ (2019). Efficacy of durvalumab in patients with stage III NSCLC who experience pneumonitis (PACIFIC). Ann Oncol.

[17] Yamada T, Uchino J, Chihara Y, Shimamoto T, Iwasaku M, Tamiya N, Kaneko Y, Kiyomi F, Takayama K (2020). Rationale and design of a phase II trial of durvalumab treatment in patients with NSCLC ineligible for stage III chemoradiotherapy following radiation monotherapy (SPIRAL-RT study). Ther Adv Med Oncol.

[18] Shaverdian N, Offin MD, Rimner A, Shepherd AF, Wu AJ, Rudin CM, Hellmann MD, Chaft JE, Gomez DR (2020). Utilization and factors precluding the initiation of consolidative durvalumab in unresectable stage III non-small cell lung cancer. Radiother Oncol.

[19] Sakaguchi T, Ito K, Furuhashi K, Nakamura Y, Suzuki Y, Nishii Y, Taguchi O, Hataji O (2019). Patients with unresectable stage III non-small cell lung cancer eligible to receive consolidation therapy with durvalumab in clinical practice based on PACIFIC study criteria. Respir Investig.

[20] Ohri N, Halmos B, Bodner WR, Cheng H, Garg MK, Gucalp R, Guha C (2020) Who benefits the Most from adjuvant Durvalumab after Chemoradiotherapy for non-small cell lung Cancer? An Exploratory Analysis. *Pract Radiat Oncol*. 10.1016/j.prro.2020.09.010

[21] Offin M, Shaverdian N, Rimner A, Lobaugh S, Shepherd AF, Simone CB, Gelblum DY, Wu AJ, Lee N, Kris MG (2020). Clinical outcomes, local-regional control and the role for metastasis-directed therapies in stage III non-small cell lung cancers treated with chemoradiation and durvalumab. Radiother Oncol.

[22] Jung HA, Noh JM, Sun J-M, Lee S-H, Ahn JS, Ahn M-J, Pyo H, Ahn YC, Park K (2020). Real world data of durvalumab consolidation after chemoradiotherapy in stage III non-small-cell lung cancer. Lung Cancer.

[23] Chu C-H, Chiu T-H, Wang C-C, Chang W-C, Huang AC-C, Liu C-Y, Wang C-L, Ko H-W, Chung F-T, Hsu P-C, Guo YK, Kuo CHS, Yang CT (2020). Consolidation treatment of durvalumab after chemoradiation in real-world patients with stage III unresectable non-small cell lung cancer. Thorac Cancer.

[24] Abe T, Saito S, Iino M, Aoshika T, Ryuno Y, Ohta T, Igari M, Hirai R, Kumazaki Y, Miura Y, Kaira K, Kagamu H, Noda SE, Kato S (2020). Effect of durvalumab on local control after concurrent chemoradiotherapy for locally advanced non-small cell lung cancer in comparison with chemoradiotherapy alone. Thorac. Cancer.

[25] Hanna N, Neubauer M, Yiannoutsos C, McGarry R, Arseneau J, Ansari R, Reynolds C, Govindan R, Melnyk A, Fisher W (2008). Phase III study of cisplatin, etoposide, and concurrent chest radiation with or without consolidation docetaxel in patients with inoperable stage III non-small-cell lung cancer: the Hoosier Oncology Group and U.S. Oncology. J. Clin. Oncol..

[26] Senan S, Brade A, Wang L-H, Vansteenkiste J, Dakhil S, Biesma B, Martinez Aguillo M, Aerts J, Govindan R, Rubio-Viqueira B, Lewanski C, Gandara D, Choy H, Mok T, Hossain A, Iscoe N, Treat J, Koustenis A, San Antonio B, Chouaki N, Vokes E (2016). PROCLAIM: randomized phase III trial of Pemetrexed-Cisplatin or Etoposide-Cisplatin plus thoracic radiation therapy followed by consolidation chemotherapy in locally advanced nonsquamous non-small-cell lung Cancer. J Clin Oncol.

[27] Flentje M, Huber RM, Engel-Riedel W, Andreas S, Kollmeier J, Staar S, Dickgreber N, Vaissiere N, de Almeida C, Edlich B, Fietkau R (2016). GILT--A randomised phase III study of oral vinorelbine and cisplatin with concomitant radiotherapy followed by either consolidation therapy with oral vinorelbine and cisplatin or best supportive care alone in stage III non-small cell lung cancer. Strahlenther Onkol.

[28] Kelly K, Chansky K, Gaspar LE, Albain KS, Jett J, Ung YC, Lau DHM, Crowley JJ, Gandara DR (2008). Phase III trial of maintenance gefitinib or placebo after concurrent chemoradiotherapy and docetaxel consolidation in inoperable stage III non-small-cell lung cancer: SWOG S0023. J Clin Oncol.

[29] Ramalingam S, Sandler AB (2006). Salvage therapy for advanced non-small cell lung cancer: factors influencing treatment selection. Oncologist.

[30] Käsmann L, Taugner J, Eze C, Roengvoraphoj O, Dantes M, Gennen K, Karin M, Petrukhnov O, Tufman A, Belka C (2019). Performance Status and Its Changes Predict Outcome for Patients With Inoperable Stage III NSCLC Undergoing Multimodal Treatment. Anticancer Res..

